# Protein post-translational modifications: key switches coordinating fruit ripening regulatory networks

**DOI:** 10.1093/hr/uhaf351

**Published:** 2025-12-09

**Authors:** Xiaojing Li, Qian Li, Guozheng Qin, Bingbing Li

**Affiliations:** Department of Pomology, College of Horticulture, China Agricultural University, Beijing 100193, China; College of Horticulture, Hebei Agricultural University, Baoding, Hebei 071000, China; Department of Pomology, College of Horticulture, China Agricultural University, Beijing 100193, China; The College of Food Science and Nutritional Engineering, China Agricultural University, Beijing 100083, China; Department of Pomology, College of Horticulture, China Agricultural University, Beijing 100193, China

## Abstract

Fruit ripening is a highly coordinated developmental process that transforms immature fruits into edible organs adapted for seed dispersal and human consumption. Although transcriptional regulation has long been acknowledged as a fundamental mechanism underlying ripening control, accumulating evidence now indicates that post-translational modifications (PTMs) function as master regulatory switches that precisely control protein activity, stability, and interactions. PTMs such as phosphorylation, ubiquitination, acetylation, redox modifications, and methylation establish dynamic regulatory networks that integrate hormonal signals, metabolic fluxes, and environmental signals to control the complex biochemical and physiological changes during fruit ripening. This review summarizes current understanding of PTM-mediated regulation in both climacteric and nonclimacteric fruits, emphasizing how modification cascades control key processes including ethylene signaling, cell wall remodeling, pigment accumulation, and stress responses. We explore emerging crosstalk networks in which multiple PTMs target important proteins to form complex molecular switches and discuss recent methodological advances that facilitate systems-level analysis of PTM. Integrating PTM research with precision agriculture and biotechnology offers promising approaches for improving fruit quality, extending shelf-life, and enhancing stress tolerance in the context of global climate change.

## Introduction

Fleshy fruits represent an evolutionary innovation for optimal seed dispersal, achieved through tightly coordinated regulatory programs that enhance nutritional content, modify texture, and develop visual appeal at precise developmental stages [1]. This complex transformation, termed ripening, integrates cell growth, cell wall remodeling, and metabolic reprogramming—processes collectively determining fruit quality and consumer preference [2]. Understanding the regulation of growth and ripening is agriculturally critical, as it precisely regulates yield, postharvest resistance, and nutritional value.

**Figure 1:**
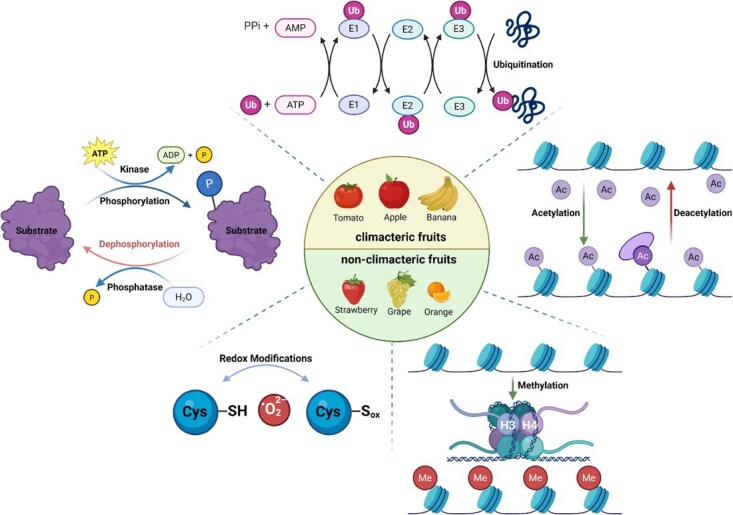
Overview of major PTMs regulating fruit ripening networks. The tripartite enzymatic system regulating protein modifications during fruit development is illustrated. The ubiquitination cascade involves E1 activating enzymes, E2 conjugating enzymes, and E3 ligases, which determine substrate specificity, utilize ATP, and generate ubiquitinated proteins. Phosphorylation is mediated by kinases that add phosphate groups using ATP, while phosphatases remove these modifications through hydrolysis. Both climacteric fruits tomato, apple, and banana and nonclimacteric fruits strawberry, grape, and orange are regulated by these modification systems. Acetylation and deacetylation processes modify lysine residues on histones and nonhistone proteins, altering chromatin accessibility and protein function. Redox modifications involve the oxidation of cysteine from the reduced (–SH) to the oxidized (–Sox) state in response to ROS. Histone methylation (Me) provides epigenetic control over gene expression during fruit ripening. These interconnected PTM networks form complex regulatory circuits that coordinate fruit ripening processes.

To unravel these regulatory mechanisms, fruits are classified into climacteric and nonclimacteric types based on their ripening patterns [3] (Fig. 1). Climacteric fruit ripening is predominantly regulated by ethylene, whose autocatalytic biosynthesis plays a central role in initiating and orchestrating ripening. Modulating ethylene biosynthesis or signaling pathways effectively controls ripening in these fruits, as demonstrated by the *Never ripe (Nr)*, *Green ripe (Gr)*, and *yellow-fruited tomato 1 (yft1)* mutants, which exhibit significantly delayed ripening due to disruptions in ethylene signal transduction, even in the presence of ethylene [4]. Representative species include tomatoes, apples, and bananas. In contrast, nonclimacteric fruits (e.g. strawberries, citrus, grapes), which differ from climacteric ones in ripening mechanisms, produce minimal ethylene, and abscisic acid (ABA) is increasingly recognized as a key regulator involved in anthocyanin and sugar accumulation [5]. However, its role as a primary regulator, analogous to ethylene in climacteric fruits, requires further validation, and the underlying regulatory network remains incompletely understood.

Traditional models of ripening control have centered on transcriptional hierarchies, particularly in the climacteric model tomato, where master regulators like RIPENING INHIBITOR (RIN) and NON-RIPENING (NOR) orchestrate genes involved in ethylene signaling, cell wall degradation, and flavor-related compounds metabolism [6, 7]. Despite these insights, transcription-focused paradigms fail to fully explain the rapid dynamics, reversibility, and signal-responsive tuning of ripening. The biological need for exquisitely timed biochemical transitions—where metabolic shifts align with developmental windows—demands regulatory mechanisms operating beyond the transcriptional timescale. Against this backdrop, post-translational modifications (PTMs) have emerged as the essential regulatory layers bridging genetic programs to physiological outcomes. PTMs include covalent chemical modifications added to proteins after synthesis, including phosphorylation, ubiquitination, acetylation, methylation, glycosylation, and redox modifications [8]. These modifications function as molecular switches that rapidly alter protein activity, subcellular localization, stability, and protein–protein interactions in response to developmental and environmental signals. Unlike transcriptional regulation, which requires hours to days for full effect, PTMs can modulate protein function within seconds to minutes, providing the temporal resolution necessary for precise ripening control.

Recent technological advances in mass spectrometry-based proteomics have revolutionized our ability to identify and quantify PTMs across entire proteomes, revealing extensive modification networks in ripening fruits. Comprehensive PTMomics studies have identified thousands of modification sites on proteins involved in hormone signaling, metabolism, and stress responses, demonstrating that PTMs create regulatory complexity far outstripping what is possible through transcription alone [9]. These discoveries challenge traditional linear models of ripening regulation and suggest that PTMs function as integrated networks where multiple modifications cooperate to create complex regulatory switches.

The agricultural significance of PTM research extends beyond fundamental biology to practical applications in crop improvement. Understanding PTM regulation offers new strategies for developing fruits with enhanced quality traits, extended shelf-life, and improved stress tolerance. Recent successes in engineering-specific PTM sites or modifying PTM enzymes demonstrate the potential for precision breeding approaches that fine-tune fruit characteristics without affecting other agronomic traits [10].

This review synthesizes current knowledge of PTM-mediated regulation in fruit ripening by examining the enzymatic machinery that writes, erases, and reads major PTM types, while providing specific examples of how PTMs control key ripening processes. We explore the emerging understanding of PTM crosstalk networks that create integrated regulatory circuits and discuss methodological advances enabling comprehensive PTM analysis. Throughout, we emphasize mechanistic insights that reveal general principles of PTM regulation while highlighting species-specific adaptations that reflect the diversity of ripening strategies across fruit types. Finally, we outline future directions for translating PTM knowledge into agricultural applications that could enhance fruit quality and production sustainability.

## Key PTM types and their enzymatic machinery

PTM regulation is mediated through tripartite enzymatic systems comprising “writers” that install modifications, “erasers” that remove them, and “readers” that recognize modified residues. This framework enables precise temporal and spatial control over protein function during fruit ripening. Current research focuses on identifying enzymatic components, mapping modification sites, and elucidating substrate recognition mechanisms.

*Phosphorylation*, a key PTM, operates through protein kinases (writers), protein phosphatases (erasers), and substrates that undergo phosphorylation (Fig. 1).

Plant protein kinases comprise several major families with distinct regulatory functions. Mitogen-activated protein kinases (MAPKs) act as central signaling modules, integrating multiple upstream signals and phosphorylating diverse substrates such as transcription factors and metabolic enzymes. Calcium-dependent protein kinases (CDPKs) directly bind calcium without the need for calmodulin, enabling rapid responses to calcium signals; CBL-interacting protein kinases (CIPKs) work with calcineurin B-like proteins (CBLs) to decode specific calcium signatures, mediating key biological processes. Sucrose nonfermenting 1-related kinases (SnRKs) integrate energy status and stress signals: SnRK1 functions as a master metabolic regulator, while SnRK2/3 subfamilies control ABA responses and osmotic stress [11, 12]. Receptor-like kinases (RLKs) form the largest kinase family, with over 600 members in *Arabidopsis*, featuring extracellular domains for perceiving environmental signals and developmental cues [13, 14].

Type 2C protein phosphatases (PP2Cs) are major phosphatase erasers, with over 80 *Arabidopsis* members, functioning as monomeric enzymes that dephosphorylate specific substrates to regulate ABA signaling, MAPK cascades, and stress responses [15]. Protein phosphatase 1 (PP1) and protein phosphatase 2A (PP2A) complexes achieve substrate specificity via diverse regulatory subunits. Identification of reader proteins remains challenging, though 14-3-3 proteins serve as major phosphorylation readers, recognizing specific phosphoserine/threonine motifs [16].

In the context of fruit ripening, research into phosphorylation-mediated regulation has advanced particularly in MAPK signaling. Meanwhile, RLK-mediated control of fruit ripening emerges as a key research frontier, as these receptors likely perceive fruit-specific developmental signals, pathogen threats, and environmental cues to coordinate ripening timing and quality traits.

*Ubiquitination* comprises E1 activating enzymes, E2 conjugating enzymes, E3 ligases, deubiquitinating enzymes (erasers), and reader proteins that recognize ubiquitinated substrates, collectively regulating protein stability and signaling (Fig. 1).

Plant ubiquitination involves diverse E3 ligase families that target specific substrates for degradation or signaling modification. RING finger E3 ligases form the largest family, with over 470 *Arabidopsis* members, using zinc coordination to transfer ubiquitin and control protein stability via direct substrate recognition or adaptor proteins [17]. Another major class, SKP1-Cullin-F-box (SCF) complexes utilize interchangeable F-box proteins as substrate recognition modules, enabling precise targeting of hormone signaling components (e.g. ethylene receptors), transcription factors, and cell cycle regulators. Cullin-RING ligase (CRL) complexes include CRL3 systems with BTB domain proteins and CRL4 systems with DDB1-DWD substrate adaptors, regulating DNA repair, chromatin modification, and developmental timing [18, 19]. U-box E3 ligases combine features of RING and HECT domains, with many members responding to stress conditions and pathogen attack [20].

Plant ubiquitin-conjugating enzymes (E2s) are highly diverse with 34–200 members across species. These enzymes determine substrate fate by forming different ubiquitin linkages through various lysine residues, regulating plant growth, development, and stress responses [21]. Deubiquitinating enzymes (DUBs) function as erasers to reverse ubiquitination: the UBP/USP family, with 27 *Arabidopsis* members, regulates gene silencing, immunity, and development, while JAMM-family metalloproteases such as RPN11 provide essential proteasome-associated deubiquitination [22]. The 26S proteasome serves as the primary reader for polyubiquitinated proteins, while autophagy receptors and ESCRT complexes recognize specific ubiquitin signals for alternative degradation pathways [23].

E3 ligase-mediated regulation of master ripening regulators is recognized as a key mechanism for fine-tuning fruit quality traits, offering opportunities for biotechnological manipulation of shelf-life, color development, and nutritional content.

*Acetylation* regulates gene expression through chromatin modification (via histones) and direct modulation of nonhistone protein function, orchestrated by the dynamic balance between histone acetyltransferases (HATs, writers) and lysine deacetylases (KDACs, erasers) (Fig. 1).

HATs comprise functionally distinct families: GNAT-family enzymes such as GCN5, HAT1, and ELP3 that acetylate core histones for transcriptional activation; MYST-family enzymes involved in DNA repair and chromatin assembly; CBP-family enzymes functioning as transcriptional co-activators; and TAFII250-family enzymes controlling basal transcription and circadian rhythms [24, 25]. Recent discoveries revealed that TCP transcription factors possess intrinsic acetyltransferase activity, directly linking transcriptional regulation with chromatin modification [26].

Conversely, KDACs counteract HAT activity through three major families: RPD3/HDA-type enzymes requiring zinc cofactors and associating with transcriptional repressor complexes, SIR2-type enzymes using NAD+ as cofactor to link acetylation with cellular metabolism, and plant-specific HD2 enzymes functioning as nucleolar phosphoproteins that regulate ribosomal RNA processing [27]. Bromodomain proteins serve as acetylation readers, including BRM chromatin remodeling complexes and BET-type transcriptional regulators that recognize specific acetylated lysine patterns on histones and nonhistone proteins.

Proteomics studies have identified extensive nonhistone acetylation, with HATs and HDACs (also known as KDACs) broadly participating in acetylation and deacetylation of nonhistone proteins, affecting cellular processes including transcription, phase separation, autophagy, and differentiation [28]. Such histone acetylation patterns at gene promoters provide epigenetic switches coordinating developmental transitions, offering biotechnological potential for manipulating ripening processes.

*Redox modifications* are orchestrated by three component categories: enzymes generating reactive oxygen species (ROS) and nitric oxide (NO) (writers), antioxidant enzymes that reverse these modifications (erasers), and redox-sensitive proteins that transduce redox signals (readers). Together, these form reversible molecular switches integrating cellular redox status with protein function in plants (Fig. 1).

ROS-generating systems include several important enzyme families, with distinct functions: NADPH oxidases such as RBOHD and RBOHF produce extracellular H₂O₂ for cell wall cross-linking and signaling; class III peroxidases, with more than 70 genes in *Arabidopsis*, participate in lignification and suberization processes [29]; lipoxygenases generate lipid hydroperoxides affecting membrane dynamics; and nitrate reductases produce NO for protein S-nitrosylation [30].

Antioxidant eraser systems maintain redox homeostasis through three major pathways, each with specialized roles: the thioredoxin system includes plastidic, cytosolic, and mitochondrial isoforms that reduce disulfide bonds using NADPH as the electron donor; the glutaredoxin system comprises dithiol GRXs (functioning in protein deglutathionylation) and monothiol GRXs (involved in iron–sulfur cluster assembly); and peroxiredoxins scavenge H₂O₂ while transmitting oxidative signals [30].

Redox modifications encompass S-nitrosylation, S-glutathionylation, sulfenylation, disulfide bond formation, persulfidation, and methionine oxidation. Redox-sensitive transcription factors like NPR1 and TGA proteins contain critical cysteine residues that undergo oxidation/reduction cycles to control DNA binding and nuclear localization [31].

Redox homeostasis shifts during fruit ripening coordinate antioxidant capacity with developmental progression, offering targets for enhancing fruit nutritional quality and postharvest preservation.

*Methylation* is mediated by methyltransferases (writers), demethylases (erasers), and reader proteins, primarily regulating transcriptional activity in plants (Fig. 1).

Plant histone lysine methyltransferases include SET domain proteins organized into functional classes with distinct methylation specificities: SU(VAR)39 groups [including SU(VAR)3–9 homologs (SUVH) and SU(VAR)3–9-related proteins (SUVR)], TRX (trithorax) groups (TRX homologs and TRX-related proteins), and ASH1 (absent, small, or homeotic discs 1) groups [ASH1 homologs (ASHH) and ASH1-related proteins (ASHR)], E(Z) (enhancer of zeste) homologs [32–35]. Protein arginine methyltransferases (PRMTs) generate asymmetric or symmetric dimethylarginine on RNA-binding proteins, transcription factors, and histone tails to modulate gene expression (e.g. via transcription factor regulation) and RNA processing (e.g. splicing and translation) [36].

Histone demethylases featuring JmjC domains remove specific methylation marks: KDM3 subfamily (IBM1) for H3K9 demethylation, KDM4 subfamily for H3K9/H3K36 demethylation, KDM5 subfamily for H3K4 demethylation, and KDM6 subfamily for H3K27 demethylation to activate gene expression [37].

Methylation readers include chromodomain proteins (LHP1 for H3K27me3 recognition), Tudor domain proteins for methylarginine binding, PWWP domain proteins for H3K36me3 recognition, and PHD finger proteins that discriminate between different histone methylation states (e.g. H3K4me3 vs. H3K27me3) to fine-tune transcriptional output [38].

Histone methylation regulates transcriptional changes, forming molecular switches that coordinate the transcriptional reprogramming required for fruit ripening. Methylation editing represents a promising biotechnological approach for developing fruits with enhanced quality, extended shelf-life, and improved stress tolerance.

## PTM regulation of key fruit ripening processes

Fruit ripening encompasses a wide range of biological processes. In recent years, research on PTM mechanisms underlying fruit ripening has primarily focused on the regulation of key quality traits, including pigment accumulation, sugar metabolism, and cell wall remodeling. This research has involved the identification of key regulatory proteins, modified substrates, and associated pathways. With respect to different types of fruit ripening, the roles of PTMs in hormone-regulated pathways have been partially elucidated: in climacteric fruits, studies on PTM involvement in ethylene-mediated ripening pathways have achieved relatively comprehensive insights; in nonclimacteric fruits, significant progress has also been made in understanding PTM mechanisms related to ABA regulation. However, investigations into PTM-mediated regulatory pathways that govern fruit size, weight, aroma, and other quality attributes remain limited. Beyond ripening-promoting hormones, the PTM mechanisms of key hormones that regulate development and indirectly influence ripening—such as indole-3-acetic acid (IAA), gibberellins (GA), and cytokinins (CK)—are still underexplored. Moreover, the antagonistic and balanced regulatory mechanisms mediated by PTMs between development-related and ripening-related hormones remain poorly understood and require in-depth investigation. The following sections provide a review of the currently identified key PTM regulatory proteins, modified substrates, and their associated pathways in fruit ripening [27, 39].

### Phosphorylation: orchestrating metabolic integration and hormone signaling

Phosphorylation serves as a central regulatory mechanism in fruit ripening, coordinating transcriptional reprogramming, metabolic changes, and stress responses through dynamic kinase and phosphatase activities. This regulatory system operates in both climacteric and nonclimacteric fruits, with conserved pathways that exhibit functional divergence critical for fruit development. Phosphorylation-mediated signaling networks regulate fruit quality traits with specificity across distinct ripening processes.

**Figure 2:**
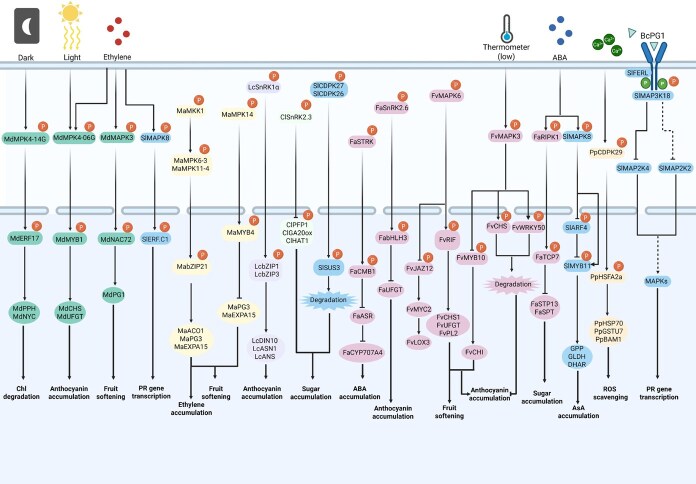
Integrated signaling networks connecting environmental cues with fruit ripening through phosphorylation cascades. Environmental inputs including dark/light transitions, ethylene signaling, temperature fluctuations, and ABA accumulation activate complex kinase networks that regulate fruit development. MAPK cascades involving MdMPK4-14G/06G, MaMPK3, and SlMPK6 phosphorylate downstream transcription factors and metabolic enzymes. Key regulatory modules include the LcSnRK1α pathway controlling energy homeostasis, ClSnRK2.3 regulating sugar accumulation, and FaSnRK2.6 modulating anthocyanin biosynthesis. Calcium-dependent kinases (CDPKs) such as PbCPK28 and FaRIPK1 integrate calcium signals with sugar transport and ABA signaling. The network converges on multiple physiological outputs: chlorophyll degradation, anthocyanin accumulation, fruit softening, pathogenesis-related gene transcription, sugar accumulation, ABA biosynthesis, and ROS scavenging. This integrated signaling framework enables precise temporal control of ripening progression in response to developmental and environmental cues.

Phosphorylation cascades control anthocyanin biosynthesis pathways, a key determinant of fruit coloration during ripening. In apple, MdMPK4-06G responds to light induction, enhancing MdMYB1 stability through phosphorylation and promoting anthocyanin biosynthesis genes *MdCHS* and *MdUFGT* expression, thereby accelerating light-induced anthocyanin accumulation [40]. Similarly, MdMPK6 mediates HY5 phosphorylation, strengthening its regulation of light-induced anthocyanin biosynthesis genes and contributing to color formation [41]. A comparable mechanism operates in strawberry, where FvMAPK6 phosphorylates the NAC transcription factor FvRIF, enhancing its transcriptional activation of target genes involved in anthocyanin biosynthesis and fruit ripening [42] (Fig. 2). Phosphorylation can also serve inhibitory functions, as demonstrated by FaSnRK2.6, which phosphorylates FabHLH3, inhibiting its binding to the *FaUFGT* promoter and suppressing anthocyanin biosynthesis [43]. Environmental stress significantly affects these pathways. Under low-temperature conditions, FvMAPK3 suppresses anthocyanin accumulation by phosphorylating FvMYB10 to reduce its activity and targeting FvCHS1 for degradation [44]. Similarly, strawberry FvWRKY50 promotes anthocyanin accumulation under normal conditions; however, under low temperature, it is phosphorylated and degraded by FvMAPK3, inhibiting pigmentation [45] (Fig. 2) (Table 1).

Beyond color formation, phosphorylation modification regulates cell wall modifications that determine fruit texture, primarily by modulating enzyme activity and gene expression. In apple, ethylene signaling induces MdMAPK3 to phosphorylate MdNAC72, enhancing its transcriptional activation of cell wall degradation genes and promoting fruit softening [46] (Fig. 2). This ethylene-responsive pathway demonstrates the integration of hormonal signaling with structural modifications essential for fruit texture. The complexity of softening regulation is further exemplified in banana: MaMKK1 activates MaMPK6-3 and MaMPK11-4, forming the “MKK1-MPK6-3/11-4” cascade. These kinases phosphorylate MabZIP21 at multiple sites, enhancing its transcriptional activation of cell wall-modifying genes *MaPG3* and *MaEXPA15*, to accelerate ripening [47, 48]. In tomato, the protein phosphatase SlPP2C2 dephosphorylates the transcription factor SlZAT5 at Ser-65, modulating its repressor activity on cell wall degradation gene *SlPL8* and thereby affecting fruit softening [49] (Fig. 2).

Phosphorylation also regulates sugar transport and metabolism, which determine fruit sweetness and sugar accumulation patterns. In pear, PbCPK28 phosphorylates the sugar transporter PbTST4 and proton pump PbVHA-A1, boosting sugar accumulation in vacuoles [50] (Fig. 2). Negative regulation of sugar accumulation provides another layer of control. In watermelon, ClSnRK2.3 negatively regulates fruit ripening and sugar accumulation by phosphorylating ClPFP1, ClGA20ox, and ClHAT1 [51]. Similarly, tomato SlCDPK27 and SlCDPK26 function as “sugar brakes” that phosphorylate SlSUS3, promoting its degradation and reducing fruit sweetness [52]. Sugar transport efficiency is enhanced through phosphorylation-mediated activation. Sweet orange CsTST2 transports sugars into vacuoles, with its activity enhanced by CsCBL1/CIPK23-mediated phosphorylation, contributing to fruit sweetness [53] (Fig. 2). Recent evidence demonstrates that phosphorylation significantly enhances SWEET transporter oligomerization and sucrose transport activity, with SnRK2-mediated phosphorylation of SWEET11/12 dramatically improving transport efficiency [54], suggesting similar mechanisms may enhance sugar accumulation in developing fruits.

**Table 1:** PTMs enzyme and substrate in fruit ripening

PTM	Species	Enzyme	Substrate	Physiology or developmental processes	Ripening stage	References
Phosphorylation	Tomato	SlPP2C2	SlZAT5	Modulating ethylene production	IMG	[49]
		SlMAPK4	SlWRKY6	Promote fruit ripening	IMG-RR	[98]
		SlCDPK27/SlCDPK26	SlSUS3	Negatively regulates fruit sugar content	MG-RR	[52]
		SlFERL	BcPG1/SlMAP3K18	Activate defense responses to *B. cinerea*	/	[128]
		SlMPK8	SlERF.C1	Enhance fruit resistance to powdery mildew	Br	[130]
	Apple	MdMPK4	MdMYB1	Enhance light-induced anthocyanin accumulation	/	[40]
		MdMPK4	MdERF17	Promotes fruit peel degreening	60–100 DAFB	[58]
		MdSnRK2.4/MdSnRK2.9	MdHB1/MdHB2	Regulate ethylene biosynthesis	60–155 DPA	[55]
		MdMAPK3	MdNAC72	Promote apple fruit softening	140 DAFB	[46]
		MdMPK6/02G	MdWRKY70L	Promote apple fruit senescence	160 DAFB	[60]
		MdMPK6	MdHY5	Enhance light-induced anthocyanin accumulation	160 DAFB	[41]
	Banana	MaMPK11–4	MabZIP21	Promote fruit ripening	MG	[47, 48]
		MaMPK14	MaMYB4	Negatively regulates fruit ripening	MG	[120]
		MaKIN10 X1/3	MaMYB13	Enhance banana fruit chilling tolerance	MG	[131]
	Peach	PpCDPK29	PpHSFA2a	Regulate postharvest chilling tolerance of fruit	RR	[132]
	Pear	PbCPK28	PbTST4/PbVHA-A1	Promote vacuolar sugar accumulation	91–120 DAFB	[50]
	Strawberry	FvMAPK6	FvRIF	Promote fruit ripening	SG-RR	[42]
		FaSnRK2.6	FabHLH3	Inhibit fruit coloration	IR-FR	[43]
		FaRIPK1	FaTCP7	Promote soluble sugar content	LG-PR	[56]
		FvMPK6	FvJAZ12	Promote flavor and defense	RR	[129]
		FaSTPK	FaCMB1	Promote ABA accumulation and fruit ripening	SG-RR	[57]
	Strawberry	FvMAPK3	FvMYB10/FvCHS1	Inhibit the accumulation of anthocyanin under low temperature	W-RR	[44]
		FvMAPK3	FvWRKY50	Delay anthocyanin accumulation at low temperature	LG-RR	[45]
	Watermelon	ClSnRK2.3	ClPFP1/ClGA20ox/ClHAT1	Negatively regulate fruit ripening and sugar accumulation	18–34 DAP	[51]
	Sweet orange	CsCIPK23	CsTST2	Enhance sugar accumulation	150–240 DAF	[53]
	Litchi	LcSnRK1a	LcbZIP1/LcbZIP3	Modulates fruit senescence	56–84 DPA	[61]
Ubiquitination	Tomato	SlPPSR1	SlPSY1	Modulate carotenoid biosynthesis in fruit	34–45 DPA	[64]
		SlUBC32	/	Regulate tomato fruit ripening	Br-Or	[63]
		SlBRG3	SlWRKY71	Delay ripening of tomato fruit	/	[92]
		SlCUL4-SlDDB1-SlDET1	SlGLK2	Regulate plastid level and fruit quality	/	[77]
		SlREM1	SlACS2/SlACO1	Accelerate fruit ripening processes	IMG-RR	[72]

**Table 1:** Continued

PTM	Species	Enzyme	Substrate	Physiology or developmental processes	Ripening stage	References
Ubiquitination	Apple	MdPUB24	MdBEL7	Promote chlorophyll degradation in fruit	ripe fruit	[75]
		MdXBAT31	MdCRF4	Amplify ethylene biosynthesis and accelerate ripening	140 DAFB	[73]
		MdBT2	MdBBX22	Regulate UV-B-induced anthocyanin biosynthesis	/	[66]
		MdBT2	MdMYB1	Negatively regulate ethylene-induced fruit coloration	120 DAFB	[134]
		MdBT2	MdbZIP44	Inhibit ABA-triggered anthocyanin accumulation	120 DAFB	[67]
	Banana	MaBRG2/MaBRG3	MaMYB4	Promote fruit ripening	MG	[68]
		MaNIP1	MaNYC1	Delay chlorophyll breakdown and peel yellowing	MG	[74]
	Pear	PuRDUF2	PuAAT1	Reduce the volatile esters during fruit storage	135 DAFB	[71]
	Papaya	CpEBF1	CpEIL1	Delay fruit ripening	MG	[69]
	Strawberry	FvCSN5A	FvPAO5	Regulate plant growth and fruit ripening	RR	[79]
	Grape	VlPUB38	VlAAO	Suppress ABA accumulation and negatively regulate fruit ripening	/	[78]
Acetylation	Tomato	SlHDA3	/	Negatively regulate fruit ripening and carotenoid accumulation	MG-Br + 7	[80]
		SlHDA7	H4ac	Suppress fruit ripening	MG-RR	[82]
		SlGAME36	hydroxylated SGAs	Converts toxic bitter α-tomatine to nonbitter, less toxic esculeoside A	LG-RR	[87]
		SlHDA1/3	H3K9ac/H3K27ac	Inhibits chlorophyll breakdown in early development	/	[83]
	Apple	MdHDT3	H3ac/H4ac	Repress ethylene biosynthesis	30–145 DAFB	[84]
		MdHDA19	H3K9	Suppress fruit ripening and ethylene production	/	[85]
	Banana	MaHDA6	H3ac/H4ac	Repress banana fruit ripening and softening	MG	[86]
		MaHDA1	H3ac/H4ac	Repress the expression of MaACO1 and expansins during fruit ripening	MG	[88]
Redox modification	Tomato	/	BRG3	Reduce ubiquitination activity and delay fruit ripening	/	[92]
		E4/SlMsrB2	SlNOR	Reduces the oxidized NOR and restore its function	39–48 DPA	[93]
		SlNTRC	SlALKBH2	Modulate m^6^A levels and fruit ripening	SG-RR	[99]
	Banana	MaMsrA4	MaEIL9	Delay fruit ripening	MG	[94]
Methylation	Tomato	SlJMJ6	H3K27	Facilitate tomato fruit ripening	Tu-RR	[100]
	Banana	MaJMJ15	H3K27me3	Positively regulate fruit ripening	MG	[101]

IMG, immature green stage IMG; RR, red ripe; MG, mature green; Br, breaker stage; DAFB, days after full bloom; DPA, day postanthesis; SG, small green; IR, initial red; FR, full red; LG, large green; PR, partial red; W, white fruit; DAP, day after pollination; DAF, day after flowering; Or, orange ripening; Tu, turning stage.

Multiple hormone signaling pathways converge through phosphorylation mechanisms that coordinate complex fruit developmental programs. Ethylene biosynthesis and signaling represent primary targets: in apple, MdSnRK2.4/MdSnRK2.9 phosphorylate HB transcription factors MdHB1/MdHB2 to enhance *MdACO1* activation and stabilize MdACS1, collectively promoting ethylene production [55]. ABA signaling in nonclimacteric fruits, exemplified by strawberry ripening, involves multiple phosphorylation-mediated regulatory modules. The receptor-like kinase FaRIPK1 mediates ABA-dependent sugar accumulation by phosphorylating TCP transcription factor FaTCP7. ABA strengthens FaRIPK1-FaTCP7 interaction and triggers FaTCP7 phosphorylation, activating sugar transporter genes *FaSTP13* and *FaSPT* to promote sucrose accumulation for fruit quality [56] (Fig. 2). ABA signaling also regulates its own homeostasis through the FaSTPK–FaCMB1 module. ABA-responsive kinase FaSTPK phosphorylates MADS-box transcription factor FaCMB1, which represses *FaASR* expression. FaASR inhibits ABA catabolic enzyme FaCYP707A4. FaSTPK-mediated FaCMB1 phosphorylation attenuates this repression, thereby activating FaCYP707A4 to degrade ABA [57]. This establishes a negative feedback loop. Elevated ABA activates FaSTPK, which acts through the FaCMB1-FaASR module to ultimately promote ABA degradation. This mechanism prevents excessive ABA accumulation and ensures proper ripening dynamics. The FaRIPK1-FaTCP7 and FaSTPK-FaCMB1 modules demonstrate how ABA-responsive kinases integrate developmental signaling with distinct outputs: FaRIPK1 links ABA to metabolic reprogramming via sugar transporters, while FaSTPK connects ABA to hormonal homeostasis via coordinated catabolism control. This phosphorylation network orchestrates metabolic and hormonal dynamics required for coordinated nonclimacteric fruit ripening.

In addition to specific quality traits, phosphorylation networks coordinate broader developmental transitions including senescence and metabolic adjustments. In apple, MdMPK4-14G activates in darkness, strengthening transcriptional activation of chlorophyll degradation genes *MdPPH* and *MdNYC* by phosphorylating MdERF17, mediating peel color change during light/dark transitions [58]. SlMAPK8 mediates antagonistic auxin and ABA regulation of ascorbic acid biosynthesis through the SlARF4–SlMYB11 module during fruit development and stress responses in tomato [59]. Senescence acceleration occurs through oxidative stress pathways, where MdMPK6-02G activates ROS-related gene expression by phosphorylating MdWRKY70L, accelerating fruit senescence [60]. Energy balance during fruit development involves SnRK-mediated pathways, as demonstrated in litchi where LcSnRK1α phosphorylates LcbZIP1 and LcbZIP3, strengthening their activation of genes like *LcDIN10*, *LcASN1*, and *LcANS*, which regulate energy balance and fruit senescence [61] (Fig. 2) (Table 1).

Recent studies in rice have revealed that OsMAPK6 phosphorylation and CLG1 ubiquitination of GW6a nonadditively enhance grain size through protein stabilization, illustrating the intricate crosstalk between phosphorylation and ubiquitination pathways in controlling organ size [62]. However, the regulatory mechanisms underlying fruit size and fruit weight remain unknown, and should be further explored in future studies.

The emerging complexity of phosphorylation pathways requires future investigations focusing on the spatiotemporal specificity of phosphorylation events across developmental stages, the evolutionary divergences of key PKs/PPs-mediated (e.g. MAPK and SnRK) pathways among fruit species, and the crosstalk between phosphorylation and other PTMs that coordinate comprehensive ripening programs.

### Ubiquitination: controlling protein stability in fruit ripening

Ubiquitination coordinates fruit ripening through precise spatial and temporal regulation of protein degradation, signal transduction, and stress adaptation. This PTM employs a hierarchical enzymatic cascade (E1–E2–E3) to regulate key ripening processes across climacteric and nonclimacteric fruits, with emerging roles in transcriptional reprogramming, hormone signaling, metabolic plasticity, and environmental resilience. The sophisticated control of fruit quality traits through ubiquitin-mediated protein turnover demonstrates remarkable specificity in coordinating developmental transitions and environmental responses.

Ubiquitination systems control pigmentation pathways by regulating protein stability of transcription factors and biosynthetic enzymes. In tomato, the MADS-box RIN transcription factor recruits E2 ubiquitin-conjugating enzymes (SIUBC32, SIUBC41) into the ripening network, where SIUBC32/41 silencing disrupts carotenoid accumulation and pigmentation [63]. Direct metabolic enzyme regulation occurs through the tomato RING-type E3 ligase PPSR1, which ubiquitinates PSY1 for proteasomal degradation, restricting carotenoid flux [64]. Apple demonstrates sophisticated negative regulation of anthocyanin biosynthesis through the BTB/TAZ domain protein MdBT2, which ubiquitinates and degrades the key transcription factor MdMYB1, acting as a potent brake on ethylene-induced fruit coloration [65]. MdBT2 also functions in environmental integration, degrading photomorphogenic transcription factor MdBBX22 under low UV conditions, while UV-B irradiation suppresses MdBT2 activity to promote pigmentation [66]. Additionally, MdBT2 targets MdbZIP44 for degradation, inhibiting ABA-triggered anthocyanin accumulation [67] (Table 1).

Fruit softening processes depend on ubiquitin-mediated degradation of cell wall enzymes and transcriptional regulators. In banana, RING-type E3 ligases MaBRG2 and MaBRG3 ubiquitinate and degrade MaMYB4, relieving its repression of cell wall-modifying genes and promoting fruit ripening [68]. In papaya, the F-box E3 ligase CpEBF1 targets CpEIL1 for degradation, while auxin-induced CpARF2 disrupts this interaction to stabilize CpEIL1 and enhance cell wall hydrolase transcription [69]. Direct regulation of cell wall enzymes provides another layer of softening control. The strawberry ABA-responsive E2 enzyme FaUBC76 stabilizes key proteins, upregulating cell wall remodeling genes and promoting fruit softening [70]. Negative regulation of softening occurs through targeted enzyme degradation. The pear U-box E3 ligase PuRDUF2 ubiquitinates GH28-family polygalacturonases for proteasomal degradation, delaying pectin solubilization and maintaining fruit firmness [71].

Ethylene biosynthesis and signaling represent primary targets of ubiquitination regulation in climacteric fruits. In tomato, the plasma membrane protein SlREM1 ubiquitinates and stabilizes ethylene biosynthesis enzymes ACS2 and ACO1, amplifying ethylene production and accelerating ripening [72]. Apple employs a Ca^2+^-responsive mechanism wherein MdXBAT31 ubiquitinates and degrades the ethylene-repressive transcription factor MdCRF4, de-repressing *MdACS1* expression to accelerate ripening [73]. Chlorophyll degradation, critical for peel color change, involves coordinated ubiquitin-mediated regulation. In banana, the E3 ligase MaNIP1 ubiquitinates chlorophyll catabolism enzyme MaNYC1 for degradation, antagonizing ethylene-mediated degreening [74]. Conversely, apple MdPUB24 promotes chlorophyll loss by degrading transcription factor MdBEL7 [75], while tomato SlPBB2 enhances this process by degrading Stay-Green proteins [76]. Developmental coordination involves integration of multiple signaling pathways through ubiquitination. In tomato, the CUL4-DDB1-DET1 E3 complex ubiquitinates chloroplast regulator SlGLK2 to coordinate plastid disassembly with ethylene responses [77], demonstrating how ubiquitination networks orchestrate complex developmental transitions.

Nonclimacteric fruits employ ubiquitination to fine-tune ABA biosynthesis and ripening progression. In grape berry, U-box E3 ligase VlPUB38 targets abscisic aldehyde oxidase (VlAAO) for proteasomal degradation, suppressing ABA accumulation and ripening [78]. VlAAO catalyzes the final ABA biosynthesis step—oxidation of abscisic aldehyde to ABA. VlPUB38-mediated VlAAO degradation creates post-translational control. High VlPUB38 activity maintains low VlAAO protein levels despite transcript accumulation, thereby preventing premature ABA synthesis. As véraison approaches, VlPUB38 declines and VlAAO accumulates. This accelerates ABA biosynthesis and initiates ripening. This creates a temporal lag between transcriptional activation and protein accumulation for precise developmental timing. Regulatory signals controlling VlPUB38 activity—developmental cues, sugar signals, or environmental inputs—remain unclear but likely involve PTMs of VlPUB38 or its adaptors.

This E3 ligase-mediated enzyme degradation controlling ABA biosynthesis is functionally analogous to ubiquitination control of ethylene biosynthesis in climacteric fruits, though molecular players differ by fruit type. In strawberry, ABA-responsive E2 enzyme FaUBC76 stabilizes regulatory proteins, upregulating *FaNCED1* and promoting ABA accumulation [70]. This contrasts with grape’s negative regulation, indicating ubiquitination can promote or suppress ABA accumulation depending on molecular context, developmental stage, and targets. Whether similar regulatory principles operate across nonclimacteric species requires further investigation.

Ubiquitination networks regulate protein turnover patterns that coordinate developmental transitions and quality trait formation during fruit ripening. Transcription factor dynamics during fruit ripening are precisely coordinated through targeted degradation or stabilization cascades that ensure proper developmental timing and environmental responsiveness. Metabolic enzyme regulation through ubiquitination is exemplified by the strawberry COP9 signalosome subunit FvCSN5A, which facilitates polyamine oxidase 5 (FvPAO5) degradation to regulate plant growth and fruit ripening [79] (Table 1). FvCSN5A interacts with FvCUL1 to target FvPAO5 for proteasomal degradation, controlling polyamine homeostasis and coordinating vegetative and reproductive development.

The sophistication of ubiquitination networks reveals key knowledge gaps requiring intensive investigation. Priority areas include elucidating E3 ligase substrate specificity mechanisms, understanding the functional significance of diverse ubiquitin chain topologies, characterizing the dynamic regulation of 26S proteasome composition during fruit development and stress adaptation, and exploring ubiquitination–metabolic enzyme integration.

### Acetylation: mediated chromatin and protein function regulation

Acetylation represents a fundamental PTM that operates through two distinct regulatory logics. Histone acetylation predominantly modulates gene expression by reconfiguring chromatin architecture. This establishes permissive or repressive chromatin states that control transcriptional accessibility. In contrast, nonhistone acetylation directly tunes enzymatic activity, protein stability, or subcellular localization. This occurs through modification of lysine residues on metabolic enzymes, transcription factors, and signaling proteins. During fruit ripening, both histone and nonhistone acetylation work coordinately to integrate transcriptional reprogramming with metabolic adjustments.

Histone acetylation regulates gene accessibility during fruit ripening, controlling temporal expression patterns of genes involved in multiple ripening pathways. HDAC-mediated repression systems keep fruits in pre-ripening states until developmental or environmental signals activate chromatin changes. In tomato, SlHDA3 and SlHDA7 maintain repressive chromatin states at promoters of ethylene biosynthesis and cell wall modification genes [80–82]. The transcription factor SlDEAR1 recruits the TPL2-HDA1/3 co-repressor complex, reducing H3K9Ac and H3K27Ac levels at the *SlSGR1* promoter to inhibit chlorophyll breakdown and maintain fruit greenness [83]. In apple, MdHDT3 suppresses ethylene biosynthesis by removing acetyl groups from H3 and H4 at *MdACS1* and *MdACO* promoters [84]. MdERF4 forms a complex with MdTPL4 and MdHDA19 to remove acetyl groups from H3K9 at the MdACS3a promoter, but a C-to-G mutation weakens this interaction, causing early ripening [85]. In banana, the MaHDA6-MaNAC154 module suppresses cell wall genes including *MaEXP1/2* and *MaPG1*, reducing H3ac/H4ac levels to delay softening [86]. Nonhistone protein acetylation extends control beyond chromatin by affecting transcription factors, metabolic enzymes, and stress proteins. In tomato, GAME36 adds acetyl groups to hydroxytomatine, converting bitter α-tomatine to nonbitter esculeoside A during ripening [87]. In banana, MaERF11 recruits MaHDA1 to remove acetyl groups from expansins, reducing enzyme activity and delaying softening [88] (Table 1).

Histone acetylation critically regulates nonclimacteric fruit ripening through ABA-responsive gene control. In strawberry, genome-wide profiling identified dynamic H3K9/K14ac and H3K27ac enrichment during development [89]. Activating acetylation marks enrich at promoters of ripening genes including *FvCYP707A4a* (ABA 8′-hydroxylase for ABA catabolism), *FvCHS* (anthocyanin biosynthesis), and *FvCEL1* (cell wall modification). H3K27ac enrichment at *FvCYP707A4a* directly regulates ABA homeostasis: increased acetylation activates transcription, enhancing ABA degradation to fine-tune temporal dynamics. This represents a direct chromatin-level mechanism linking histone acetylation to ABA metabolism in nonclimacteric fruits. Coordinate enrichment on *FvCHS* demonstrates simultaneous regulation of multiple ABA-responsive programs through integrated chromatin remodeling.

In grape, histone deacetylase HDAC19 interacts with ERF4 and is recruited to the *MYB5a* promoter, removing acetyl groups to establish repressive chromatin that inhibits anthocyanin accumulation [90]. While not directly linked to ABA signaling, ERF proteins integrate multiple hormonal inputs including ABA, suggesting potential crosstalk between deacetylation and ABA-mediated ripening. In citrus, genomic analysis identified 50 CsHATs and 16 CsHDACs, with CsHAF1/CsHAF2 peaking at the mature stage and CsHDA5 increasing throughout development [91]. These expression patterns implicate histone acetylation enzymes in citrus fruit maturation. However, specific targets—particularly ABA biosynthesis genes (*CsNCED*) or signaling components (CsPP2Cs, CsSnRK2s, CsABFs)—remain uncharacterized. Loss-of-function studies are needed to determine whether histone acetylation directly controls ABA-dependent ripening pathways in citrus as it does in strawberry.

These findings demonstrate histone acetylation regulates nonclimacteric ripening through at least two mechanisms: (1) direct control of ABA metabolic genes (*FvCYP707A4a*), modulating ABA homeostasis; and (2) coordinated regulation of ABA-responsive developmental genes, creating permissive chromatin states. Identifying ABA signaling components (PYR/PYL receptors, PP2C phosphatases, SnRK2 kinases, ABF transcription factors) as direct acetylation targets represents an important future direction for understanding chromatin-ABA pathway integration during nonclimacteric fruit ripening.

### Redox modifications: environmental signal integration

Redox modifications enable fruits to integrate environmental stress signals with developmental progression, ensuring that ripening occurs under optimal conditions while maintaining stress tolerance. WRKY transcription factors undergo persulfidation (sulfur addition) that changes their ability to repress target genes. In tomato, WRKY71 functions as a repressor of fruit ripening by binding to the promoter of *CYANOALANINE SYNTHASE1* (*CAS1*). H₂S-mediated sulfur addition increases its binding strength and enhances its ability to block gene expression [92]. NAC transcription factors are modified through methionine oxidation and restored by methionine sulfoxide reductases (Msrs). These enzymes dynamically control transcriptional activity by reversing oxidative damage. In tomato, the master ripening regulator NOR undergoes oxidation during ripening, which reduces its ability to bind DNA and activate ripening-related genes [93]. Ethylene signaling involves EIN3/EIL transcription factors that undergo reversible redox modifications, which regulate their DNA-binding capacity and transcriptional activity. In banana, MaEIL9 provides a clear example of this regulation. Methionine oxidation impairs its DNA-binding ability, while MaMsrA4-mediated reduction restores normal function [94].

Redox homeostasis undergoes significant reorganization during nonclimacteric fruit ripening, with ROS accumulation temporally coinciding with ABA-mediated developmental transitions, suggesting tight integration between redox signaling and ABA pathways. In grape berry, when ABA accumulation initiates color change and softening, it is characterized by a transient burst of H_2_O_2_ in skin cells and singlet oxygen (^1^O_2_) generation within chloroplasts, accompanied by dynamic changes in catalase activity [95]. This ROS burst precedes the major increase in anthocyanin accumulation and occurs simultaneously with peak ABA biosynthesis gene (*VvNCED1*) expression, indicating that oxidative signals may trigger or amplify ABA-mediated ripening initiation. The molecular mechanisms linking this ROS burst to ABA signaling remain unclear: potential targets include redox-sensitive ABA biosynthesis enzymes (NCED, AAO), ABA receptors (PYR/PYL proteins containing reactive cysteine residues), or ABA-responsive transcription factors (ABFs with redox-sensitive DNA binding domains). In climacteric fruits, ROS directly modifies ethylene signaling components through persulfidation, sulfenylation, and methionine oxidation; whether analogous redox modifications regulate ABA pathway components in grape and other nonclimacteric fruits represents a critical knowledge gap.

In strawberry, proteomic analysis during fruit ripening revealed coordinate upregulation of multiple antioxidant systems, including superoxide dismutase [Cu–Zn], glutathione S-transferases (GSTs), and aldo-keto reductases (AKRs) [96]. The increased abundance of these ROS-scavenging enzymes suggests active redox homeostasis control during ABA-mediated ripening. Importantly, AKRs have been implicated in both ROS detoxification and hormone metabolism, raising the possibility that they may influence ABA catabolism or conjugation. GSTs, beyond their antioxidant function, can modify hormone activity through conjugation; whether strawberry GSTs catalyze glutathione conjugation of ABA or its metabolites—thereby modulating ABA bioactivity—remains unexplored but represents an intriguing hypothesis linking redox metabolism to ABA homeostasis. Heat treatment of strawberry fruits, which delays ripening and reduces anthocyanin accumulation, affects oxidative metabolism pathways [97], suggesting that manipulating redox status can influence ABA-dependent developmental progression, though the molecular mechanisms remain uncharacterized.

The parallel occurrence of ROS dynamics and ABA accumulation during nonclimacteric fruit ripening strongly suggests redox-ABA crosstalk, yet no redox-modified ABA signaling proteins have been identified. In climacteric fruits, specific redox modifications have been characterized: persulfidation of SlWRKY6 in tomato creates a regulatory switch that modulates ripening timing [98], and methionine oxidation of MaEIL9 in banana impairs DNA binding [94]. Whether analogous redox modifications regulate ABA-responsive transcription factors (ABFs, MYBs, NACs) in nonclimacteric fruits is completely unknown. Priority research directions include the following: (1) redox proteomics to identify cysteine- or methionine-modified proteins in strawberry and grape during ripening, with particular focus on ABA pathway components; (2) functional analysis of redox modifications on recombinant ABA signaling proteins (PYR/PYLs, SnRK2s, ABFs) to determine whether oxidation, S-nitrosylation, or persulfidation modulates their activities; (3) and genetic manipulation of ROS homeostasis (e.g. overexpression or knockdown of *RBOH*, *SOD*, *CAT*) to determine causal relationships between redox status, ABA accumulation, and ripening progression in nonclimacteric fruits. Such studies would reveal whether redox modifications create ABA-environment integration mechanisms analogous to the redox-ethylene crosstalk characterized in climacteric species.

Beyond transcription factors, redox modifications also regulate epigenetic modifying enzymes. In tomato, H₂O₂-mediated oxidation of the m^6^A demethylase SlALKBH2 enhances protein stability and demethylation activity, fine-tuning fruit ripening [99] (Table 1).

Redox modifications as environmental signal integrators highlight critical research frontiers in fruit biology. Future work should prioritize understanding the reversibility mechanisms of persulfidation and S-nitrosylation, characterizing the spatial organization of redox signaling networks, and integrating redox modifications with stress tolerance for developing climate-resilient varieties.

### Methylation: maintaining gene expression states and protein function

Methylation functions through distinct mechanisms depending on substrate type. Histone methylation predominantly establishes long-term epigenetic memory by reconfiguring chromatin architecture, maintaining gene expression states across cell divisions and developmental transitions. Conversely, nonhistone methylation directly regulates protein function by modulating enzymatic activity, protein–protein interactions, or subcellular localization, often through modification of specific lysine or arginine residues on regulatory proteins and metabolic enzymes. Both regulatory modes contribute to fruit ripening control. Histone methylation provides long-term epigenetic memory that maintains gene expression states across cell divisions and developmental transitions. The dynamic regulation of H3K27me3 marks represents a major mechanism for controlling ripening gene accessibility. In tomato, JmjC-domain demethylases such as SlJMJ6 function as key activators by removing H3K27me3 from critical loci, directly targeting and activating master regulators such as RIN, ethylene biosynthesis genes such as *ACS4* and *ACO1*, and cell wall degradation enzymes such as PL and TBG4 [100]. In banana, MaJMJ15 similarly activates ripening through H3K27me3 demethylation at NAC transcription factor and ethylene synthesis gene promoters [101]. Nonhistone methylation affects enzyme activity and protein interactions beyond chromatin effects. Tomato proteome-wide analysis identified 241 lysine methylation sites on 176 proteins including thioredoxin, glutathione S-transferase, and NADH dehydrogenase [102] (Table 1).

Histone methylation, particularly repressive H3K27me3, regulates nonclimacteric fruit development and postharvest biology. In strawberry, H3K27me3 is dynamically regulated during ripening and responsive to storage conditions. During cold storage, H3K27me3 levels increase globally, correlating with downregulation of genes for aroma volatile production and color development [103]. This cold-induced repressive methylation represents how environmental stress suppresses ripening gene expression, potentially including ABA-responsive genes driving anthocyanin biosynthesis and aroma formation. Identifying genes acquiring H3K27me3 during cold storage—particularly ABA signaling components or ABA-responsive structural genes—would clarify histone methylation’s role in cold storage effects on fruit quality.

In grape berry, three H3K27 methyltransferases (VvH3K27-1/2/3) show distinct developmental expression patterns, with some increasing during ripening while others decline, suggesting stage-specific target gene methylation [104]. H₂O₂ treatment—which influences ABA accumulation and ripening—affects these methyltransferases’ expression, indicating crosstalk between redox signaling, histone methylation, and ABA-mediated ripening. However, direct target genes remain unknown. In tomato, H3K27me3 represses ripening regulators including RIN and ethylene biosynthesis genes (*ACS2*, *ACS4*, *ACO1*) until SlJMJ6-mediated demethylation [100]. Whether analogous repression occurs at ABA biosynthesis (*NCED*, *AAO*), signaling (*PYR/PYL*, *SnRK2*, *ABF*), or responsive structural genes in nonclimacteric fruits requires investigation.

### Glycosylation: modulating fruit quality and environmental resilience

Glycosylation regulates fruit ripening through mechanisms controlling gene expression, hormone responses, enzyme activities, and stress tolerance. At the gene expression level, glycosyltransferase activity coordinates ripening processes. In tomato, NSGT1 converts cleavable diglycosides into noncleavable triglycosides, preventing volatile release and thus controlling aroma development [105].

Hormone-mediated regulation involves SlSGT4, which increases under stress conditions to maintain membrane stability [106]. Enzyme control occurs through sterol glycosyltransferases (SlSGT1-4) that adjust membrane properties and build up aroma precursors in grapes, with environmental conditions affecting these activities [107].

Stress response mechanisms involve increased glycosylation under osmotic and cold stress, preserving cell structure while allowing continued ripening. Glycosylation also functions in plant defense against pathogens [108]. Beyond ripening, protein glycosylation plays important roles in early fruit development, as shown by CLV3 sugar modifications essential for proper development and fruit chamber control [109].

The multifaceted roles of glycosylation reveal promising directions for understanding fruit quality determination. Key areas include systematic glycoproteomics approaches to elucidate modification consequences, engineering glycosylation pathways for enhanced sensory profiles, and characterizing environmental responsiveness for developing stress-tolerant varieties.

### Emerging PTM networks: expanding the regulatory landscape

Several additional PTMs have emerged as complementary regulatory mechanisms that fine-tune fruit ripening through distinct molecular pathways. Protein lipidation, particularly ATG8 lipidation, controls fruit ripening through autophagy modulation. ATG8 lipidation involves conjugation of phosphatidylethanolamine to ATG8 proteins, forming complexes essential for autophagosome formation. Studies in strawberry revealed two distinct waves of enhanced ATG8 lipidation during ripening at white and ripe stages [110]. In tomato, SlATG8f overexpression enhances protein lipidation during the breaker stage, leading to increased autophagosome formation and accelerated fruit ripening [111].

Proteolytic processing represents a critical regulatory mechanism controlling fruit ripening through precise modulation of enzyme activities and transcription factor stability. Vacuolar processing enzymes, particularly SlVPE3 in tomato, orchestrate multiple ripening processes including pigmentation, lycopene biosynthesis, and ethylene production, with the RIN transcription factor directly regulating *SlVPE3* expression [112]. Additionally, cell wall-degrading enzymes such as polygalacturonase, β-galactosidase, and pectin methylesterase undergo specific cleavage to achieve mature, active forms during fruit softening [113].

Nitro modifications, particularly S-nitrosylation and tyrosine nitration, modulate protein function through reversible and irreversible chemical modifications. In pepper, fruit ripening involves enhanced protein tyrosine nitration, with catalase as the most abundant nitrated protein, while exogenous nitric oxide treatment delays ripening [114]. In tomato, NO-mediated modifications trigger extensive transcriptional and metabolic changes affecting one-third of the fruit transcriptome, enhancing nutritional quality with 25% increased ascorbate and 60% elevated flavonoid content [115].

Lactylation and crotonylation modifications remain largely unexplored in fruit biology. While lactylome profiling in rice identified 638 lysine lactylation sites in metabolic proteins [116], fruit studies are lacking. Crotonylation studies in papaya revealed 5995 lysine sites across 2120 proteins, including essential ripening regulators such as ethylene biosynthesis enzymes and cell wall-modifying enzymes [117].

Emerging PTM networks open new frontiers with profound implications for plant-specific regulatory mechanisms. Critical directions include characterizing the unique properties of ATG8 lipidation in plants, investigating metabolite-driven PTMs linking metabolism to protein function, and developing systems biology approaches for integrating diverse PTM networks into comprehensive regulatory models.

## PTM crosstalk networks in fruit ripening regulation

PTM crosstalk functions as a complex regulatory mechanism coordinating fruit ripening through dynamic, multilayered protein modifications [9]. Recent advances demonstrate that fruit ripening is controlled by integrated networks where different PTMs operate cooperatively rather than independently, creating combinatorial regulatory complexity that enables precise temporal and spatial control of ripening processes [118]. This regulatory model fundamentally transforms our understanding of fruit development by integrating classical hormonal signaling pathways with specific molecular switches, allowing fruits to respond dynamically to developmental cues and environmental conditions through layered regulatory circuits [119].

### Phosphorylation–ubiquitination regulatory circuits

Sequential modification events represent the most characterized form of PTM crosstalk, where initial PTMs serve as recognition signals for subsequent modifications [118]. This principle is demonstrated by phosphorylation-dependent ubiquitination cascades, where protein kinase-mediated phosphorylation creates recognition sites for E3 ubiquitin ligases. These mechanisms reveal that PTM crosstalk operates through temporal “modification timing codes.” In this system, sequential modifications create precise regulatory control. This temporal hierarchy enables fine-tuned responses to environmental and developmental signals during fruit ripening.

**Figure 3:**
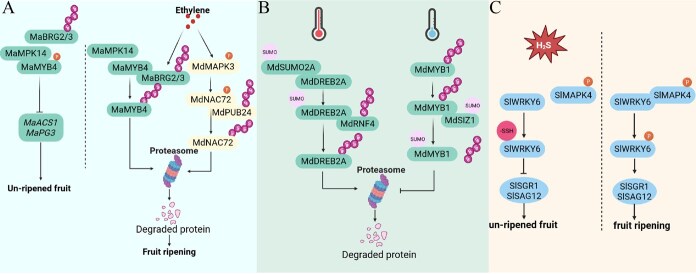
PTM crosstalk networks create complex molecular switches controlling fruit ripening. (A) Phosphorylation–ubiquitination crosstalk operates through opposite regulatory logics to control transcription factor stability. In banana fruit, MaMPK14-mediated phosphorylation of MaMYB4 provides protective function: phosphorylated MaMYB4 (left, unripened fruit) is shielded from E3 ligase-mediated ubiquitination, maintaining transcription factor stability and activity. During ripening progression (upper right), decreased MaMPK14 activity removes this protective phosphorylation, allowing MaBRG2/3 to ubiquitinate MaMYB4 for proteasomal degradation, thereby promoting fruit ripening. Conversely, in apple (lower right), ethylene-induced MdMAPK3 phosphorylates MdNAC72, creating a recognition signal for E3 ligase MdPUB24-mediated ubiquitination and degradation. These contrasting mechanisms—protective versus facilitative phosphorylation—demonstrate how the same PTM crosstalk can achieve opposite regulatory outcomes depending on molecular context. (B) Temperature-responsive SUMOylation–ubiquitination competition modulates protein stability. Under low-temperature conditions, MdSUMO2A SUMOylates MdDREB2A at K192, while under high temperature, MdDREB2A is recognized by ubiquitin E3 ligase MdRNF4 for proteasomal degradation. SUMO E3 ligase MdSIZ1 promotes anthocyanin accumulation by SUMOylating MdMYB1, which enhances protein stability and prevents ubiquitin-mediated degradation. The SUMO–ubiquitin switch enables temperature-sensitive regulation of stress responses. (C) Redox–phosphorylation crosstalk integrates environmental stress signals with ripening control. H₂S-mediated persulfidation of SlWRKY6 delays fruit ripening by suppressing target gene expression, while SlMAPK4-mediated phosphorylation promotes ripening progression. The competitive modification of SlWRKY6 creates a regulatory switch that fine-tunes ripening timing in response to cellular redox status.

The phosphorylation–ubiquitination crosstalk creates temporal control mechanisms fundamental to fruit ripening progression. In banana fruit, the MaMYB4–MaMPK14 regulatory circuit demonstrates reciprocal crosstalk where MaMPK14 phosphorylates MaMYB4 at Ser160, enhancing DNA-binding ability and protein stability while protecting MaMYB4 from ubiquitin-mediated degradation by E3 ligases MaBRG2/3 [120] (Fig. 3A). During ripening progression, decreased MaMPK14 levels allow MaBRG2/3 to ubiquitinate and degrade MaMYB4, controlling expression of ripening-associated genes including *MaACS1*, *MaPG3*, and *MaXTH5*. Apple fruit extends this regulatory logic through convergent regulation of MdbHLH3, which integrates glucose and ethylene signaling through dual PTM inputs [121] (Fig. 3A). MdHXK1 phosphorylates MdbHLH3 in response to glucose signals, while MdPUB29 ubiquitin ligase targets the same protein for degradation as part of ethylene pathway regulation [122]. Beyond anthocyanin regulation, this crosstalk controls fruit texture modifications. Ethylene enhances MdMAPK3-mediated phosphorylation of MdNAC72 to promote apple fruit softening, where MdMAPK3 phosphorylates the transcriptional repressor MdNAC72, which is subsequently ubiquitinated by MdPUB24 and degraded via the 26S proteasome pathway, demonstrating how the ethylene-MdMAPK3-MdNAC72-MdPUB24 module controls climacteric fruit ripening [46] (Fig. 3A). These regulatory circuits demonstrate “protective phosphorylation” where phosphorylation shields key ripening proteins from degradation rather than targeting them for destruction. This mechanism appears fundamental to fruit biology, enabling stable protein function during the massive cellular reorganization of ripening.

### SUMOylation and additional PTM crosstalk networks

**Figure 4:**
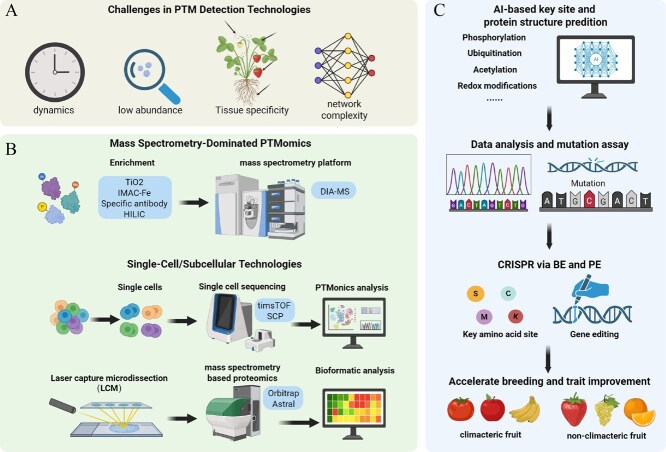
Integrative pipeline combining PTM detection technologies with AI-driven crop improvement strategies. (A) Four fundamental challenges limit PTM detection in fruit tissues: rapid modification dynamics during development, low stoichiometric abundance of many PTMs, tissue-specific modification patterns, and complex interactions within regulatory networks. These technical limitations require specialized analytical approaches for accurate PTM characterization. (B) Mass spectrometry-based PTMomics uses targeted enrichment strategies including TiO₂-based phosphopeptide capture, IMAC-Fe affinity chromatography, modification-specific antibodies, and HILIC chromatography, followed by high-resolution DIA-MS analysis. Single-cell and subcellular technologies represent emerging frontiers, utilizing single-cell sequencing with timsTOF SCP instruments for PTMomics analysis at cellular resolution. LCM enables tissue-specific sampling, combined with Orbitrap Astral mass spectrometry for comprehensive proteome-wide PTM identification. Integrated bioinformatic analysis platforms process complex datasets to clarify PTM networks and functional relationships in fruit ripening systems. (C) AI-based algorithms predict key modification sites for phosphorylation, ubiquitination, acetylation, and redox modifications across target proteins. Data analysis integrates computational predictions with experimental mutation assays to identify functionally critical amino acid residues (serine, cysteine, methionine, lysine). CRISPR-mediated base editing (BE) and prime editing (PE) technologies enable precise modification of key sites in target genes. This integrated approach accelerates breeding programs and trait improvement in both climacteric fruits (tomato, banana) and nonclimacteric fruits (strawberry, grape, orange), facilitating the development of cultivars with enhanced ripening control, extended shelf-life, and improved nutritional quality.

The crosstalk between SUMOylation and ubiquitination represents a complex regulatory network that fine-tunes protein stability and functional activity in response to developmental cues and environmental stresses. In drought stress responses, the apple MdSUMO2A-mediated SUMOylation of MdDREB2A at K192 serves as a molecular tag, facilitating its recognition by the RING-type ubiquitin E3 ligase MdRNF4, which triggers K48-linked ubiquitination and proteasomal degradation [123] (Fig. 3B). Conversely, under low-temperature conditions, the SUMO E3 ligase MdSIZ1 promotes anthocyanin accumulation by SUMOylating MdMYB1, which enhances protein stability and prevents ubiquitin-mediated degradation. This SUMOylation modification enables MdMYB1 to maintain its transcriptional activity, directly promoting the expression of anthocyanin biosynthetic genes including *MdDFR* and *MdANS* [124] (Fig. 3B). This competitive network enables “environmental fate switching” where external conditions determine protein stability or degradation fates. The SUMOylation–ubiquitination balance functions as a molecular stress memory system that influences fruit responses to environmental changes (Fig. 4).

SUMOylation also engages in crosstalk with phosphorylation and redox modifications to create integrated regulatory networks. In *Arabidopsis*, the transcription factor CESTA undergoes CDPK-mediated phosphorylation that inhibits its SUMOylation, thereby modulating brassinosteroid signaling—a mechanism potentially relevant to fruit texture modification during ripening [125]. This phosphorylation–SUMOylation antagonism represents a general regulatory logic where phosphorylation creates a “molecular shield” blocking SUMOylation sites, enabling dynamic switching between modification states. Redox modifications add another regulatory dimension. The SUMO-conjugating enzyme SCE1 in *Arabidopsis* undergoes S-nitrosylation upon pathogen exposure, inhibiting global SUMOylation [126]. Whether similar redox–SUMOylation coupling operates during fruit ripening—when oxidative stress increases and cellular redox status shifts—could link environmental stress signals with developmental progression. Large-scale analyses in *Arabidopsis* also revealed coordinated diurnal oscillations in phosphorylation and acetylation of metabolic enzymes [127], suggesting that temporal PTM patterns may coordinate ripening metabolism with circadian rhythms. These conserved crosstalk patterns, though characterized primarily in model systems, likely operate in fruits to create multilayered regulatory networks where SUMOylation functions as an integration hub. In fruit systems, redox–phosphorylation crosstalk has been directly demonstrated: H₂S-mediated persulfidation of tomato SlWRKY6 attenuates its phosphorylation by SlMAPK4, creating a regulatory switch that modulates ripening timing [98]. This represents a rare example of experimentally validated crosstalk between emerging redox modifications and classical phosphorylation in fruits. Beyond these characterized interactions, additional emerging modifications including protein lipidation [110, 111], proteolytic processing [112, 113], nitrosylation [114, 115], lactylation [116], and crotonylation [117] have been detected during fruit ripening. However, their potential crosstalk with classical PTMs remains largely unexplored, representing important future research directions for understanding the complete PTM regulatory landscape controlling fruit development.

### PTM-mediated environmental stress adaptation

Environmental stress adaptation during fruit development relies on sophisticated PTM networks that integrate stress perception with protective responses while maintaining normal ripening progression. Phosphorylation-mediated signaling cascades serve as primary stress response mechanisms through pathogen resistance and temperature tolerance pathways. In tomato, the receptor-like kinase SlFERL recognizes the Botrytis cinerea virulence protein BcPG1 and phosphorylates SlMAP3K18, which affects SlMAP2K2/4 to mediate immune responses [128]. Similarly, strawberry FvMPK6 phosphorylates FvJAZ12, reducing its nuclear accumulation to de-repress FvMYC2 and upregulate FvLOX3, promoting flavor compounds and defense responses [129]. An ethylene-MPK8-ERF.C1 module enhances resistance to B. cinerea, where ethylene induces SlMPK8, which phosphorylates SlERF.C1 to activate pathogenesis-related genes without compromising fruit ripening [130]. Temperature stress tolerance involves multiple phosphorylation pathways, as demonstrated by banana transcription factor MaMYB13, which is induced by chilling stress and activates genes involved in fatty acid and phenylpropanoid biosynthesis, with its activity enhanced by MaKIN10 X1/3 phosphorylation [131]. Hot water treatment enhances peach fruit chilling tolerance via PpCDPK29, which maintains ROS balance by interacting with PpRBOHC/D and PpSOD/PpCAT1, and phosphorylates PpHSFA2a, promoting its nuclear movement to activate stress defense genes [132].

Complementing phosphorylation networks, ubiquitination-mediated systems provide additional layers of stress integration through targeted protein degradation and stabilization. Hydrogen sulfide signaling demonstrates post-translational crosstalk complexity, where H₂S signaling reduces BRG3 ubiquitination activity through persulfidation, decreasing WRKY71 degradation and delaying ripening in tomato [92]. Multiple environmental stress responses converge through specific E3 ligases, with apple MdBT2 functioning as a central hub integrating stress signals by degrading drought-responsive MdERF38, wound-responsive MdWRKY40, and high light-responsive MdTCP46 [133, 134]. Proteostasis maintenance during stress represents a fundamental function of ubiquitination networks, as the ubiquitin-proteasome system clears misfolded proteins during oxidative and thermal stress, exemplified by heat-inducible MaNIP1 modulating chlorophyll degradation in banana [74]. These integrated PTM networks demonstrate that fruit stress adaptation operates through coordinated molecular switches that balance developmental progression with environmental resilience, suggesting that understanding these mechanisms could inform breeding strategies for climate-adapted fruit varieties.

These conserved crosstalk patterns, though not yet characterized in fruits, hold potential to fine-tune ripening-related transcription factors, stress responses, and metabolic pathways, making them promising targets for future research. These diverse PTM crosstalk networks demonstrate hierarchical regulation, where acetylation, methylation, and other PTMs serve as modulators of core phosphorylation–ubiquitination pathways. This layered regulatory architecture suggests that fruit ripening control operates through multiple levels of modification crosstalk, each contributing distinct regulatory precision to the overall ripening program.

## Methodological frontiers: studying PTMs in fruit ripening

The rapid advancement of PTM detection technologies has fundamentally transformed our understanding of fruit ripening. Rather than viewing modifications as isolated events, we now recognize that ripening is orchestrated by dynamic, interconnected modification networks. This section discusses how technological breakthroughs have driven biological discoveries, remaining challenges, and emerging approaches.

Mass spectrometry-based PTMomics has evolved from identifying individual PTM sites to mapping entire modification landscapes during fruit development. Comprehensive phosphoproteomics in tomato identified 4011 phosphorylation sites and revealed that master regulators like RIN and NOR undergo stage-specific phosphorylation that modulates their activity—a regulatory layer invisible to gene expression studies alone [135]. Similarly, ubiquitinome profiling in papaya uncovered 3090 modification sites on 1249 proteins, demonstrating that protein degradation is remarkably selective with specific enzymes targeting distinct substrates at precise ripening stages [136]. Redox proteomics in tomato identified 70 cysteine-containing peptides from 51 oxidation-sensitive proteins that integrate environmental stress with developmental programs [137]. Despite this progress, technical limitations persist. The most significant challenge is detecting modifications occurring at very low levels—many functionally critical PTMs are present on less than 1% of total protein molecules yet govern key regulatory decisions. Advances in enrichment strategies have improved detection sensitivity [138], but dynamic changes during rapid developmental transitions remain challenging to capture. Tissue heterogeneity further complicates matters, as fruit epidermal cells and mesocarp cells have vastly different PTM profiles, yet most studies analyze bulk tissue, averaging signals across functionally distinct cell populations.

Single-cell and subcellular PTMomics represent the next major advance for addressing spatial heterogeneity. Plant cell walls must be enzymatically digested for cell isolation, which can artificially trigger stress-responsive modifications during sample preparation. Nevertheless, proof-of-concept studies have detected thousands of proteins per plant cell [139, 140], suggesting that cell-type-specific PTM profiling in fruits is technically feasible. Laser capture microdissection (LCM) offers an alternative strategy, enabling tissue-specific analysis without enzymatic stress, though sacrificing single-cell resolution. Emerging spatial profiling technologies have revealed unexpected patterns [141]. For instance, metabolic enzymes show heterogeneous acetylation across tomato pericarp regions, suggesting that seemingly uniform tissues actually employ spatially compartmentalized regulation. The biological implications are profound: ripening may not be a synchronized, tissue-wide process as commonly assumed, but rather a spatially coordinated wave with different cell layers undergoing PTM-mediated transitions at precisely timed intervals.

Integrating PTMomics with other analytical approaches has provided crucial insights into how different regulatory layers interact. Studies in apple showed that high nitrogen conditions suppress flavonoid accumulation not through reduced gene expression, but through acetylation-mediated enzyme inactivation [142]. Similarly, work in strawberry demonstrated that PTM patterns, rather than gene expression levels, determine metabolic flux distribution across different fruit tissues [143]. The key insights is that different regulatory layers operate on distinct timescales: transcriptional changes establish cellular potential over hours to days, while PTMs provide real-time adjustment within minutes to hours. This creates a robust control system where transcription sets baseline capacity while PTMs dynamically adjust activity in response to immediate signals.

Precision genome editing has moved PTM research from correlation to causation, enabling direct functional testing of specific modification sites. Base editing and prime editing enable single-nucleotide changes at specific PTM sites without broader genomic damage [144–147]. Recent technical improvements have enhanced editing efficiency in multiple crop species [148]. This precision is critical for testing PTM function: converting a phosphorylation site to an unmodifiable residue, or to one mimicking permanent phosphorylation, directly tests whether that specific modification controls a particular trait. Importantly, transgene-free editing approaches have been successfully demonstrated [149], enabling genetic improvements without foreign DNA integration—an important consideration for regulatory approval and public acceptance. Combining artificial intelligence with genome editing has created a powerful discovery platform. Structure prediction algorithms identify candidate PTM sites based on protein architecture, evolutionary conservation, and functional context, which prime editing then validates [150, 151]. A recent example demonstrated this potential: researchers combined AI-predicted modification sites with targeted protein engineering to develop citrus varieties resistant to a devastating disease [152]. Early agricultural successes validate this approach—targeted editing of phosphorylation sites increased fruit sweetness without reducing yield [153], modifications to ethylene pathway PTM sites extended shelf-life [154], and engineering PTM sites in source-sink regulatory pathways conferred enhanced yield under normal conditions while maintaining productivity under heat stress [155].

Despite these advances, fundamental gaps constrain comprehensive understanding of PTM regulation in fruit ripening. Most PTMomics studies focus on one modification type, yet crosstalk between modifications is the dominant regulatory mode. Methods simultaneously profiling multiple PTM types on the same proteins would reveal crosstalk mechanisms currently only inferred. The research community has focused heavily on tomato, apple, and grape, creating knowledge gaps for tropical fruits and other economically important species [156]. Extending PTMomics to diverse crops would reveal whether mechanisms discovered in model fruits represent universal principles or species-specific adaptations—fundamental biology with practical implications for crop improvement.

## Conclusions and future perspectives

**Figure 5:**
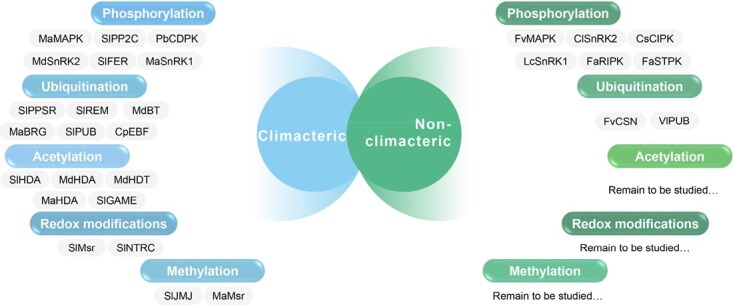
Comprehensive PTM regulatory framework governing fruit ripening across climacteric and nonclimacteric species.

The past decade has fundamentally transformed our understanding of fruit ripening regulation. PTMs have emerged as master regulatory switches integrating genetic programs, environmental cues, and metabolic status in both climacteric and nonclimacteric fruits (Fig. 5). Several core principles are now established: modification cascades enable signal amplification and temporal control; sequential modifications create molecular timers determining protein fate; combinatorial switches integrate complex signals through multiple PTMs on single proteins; and hierarchical organization generates regulatory modules with distinct kinetic properties—phosphorylation enabling rapid and reversible regulation, ubiquitination mediating protein turnover, and methylation establishing stable epigenetic memory. Importantly, ripening strategy diversity reflects distinct PTM network architectures: ethylene-centric cascades dominate climacteric fruits, while ABA-mediated networks characterize nonclimacteric species [157].

Despite remarkable progress, fundamental biological questions remain unanswered. The “reader problem” represents a critical gap: while we increasingly understand PTM writers and erasers, the proteins recognizing modified residues and converting them into functional outputs remain largely unknown in plants [158]. The “specificity paradox” poses another challenge: the mechanisms by which modification systems achieve substrate specificity when hundreds of proteins undergo similar modifications remain poorly characterized. The “network architecture question” concerns whether PTM networks exhibit modularity—whether distinct subnetworks controlling pigmentation, softening, or metabolism can be independently tuned, or whether they are inseparably linked. Understanding these system-level properties would enable predictive engineering of ripening traits.

Evolutionary perspectives offer unexplored insights into PTM network origins and diversification. The divergence of PTM regulatory architectures between climacteric and nonclimacteric lineages remains poorly understood. Certain modifications may represent molecular targets of human selection, unique to domesticated varieties. Comparative studies across wild relatives and cultivated varieties could reveal how artificial selection shaped post-translational regulation, potentially identifying “domestication PTMs” that trade stress tolerance for fruit quality—knowledge applicable to reintroducing stress resilience into elite varieties.

**Figure 6:**
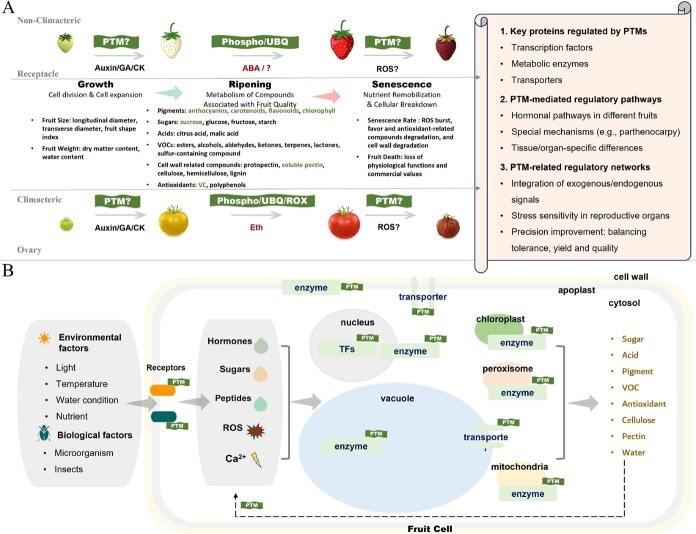
Proposed research roadmap of the PTM-mediated regulatory framework in fruit development and ripening. (A) Schematic illustration of PTM-involved regulatory mechanisms in fruit growth, ripening, and senescence. It compares nonclimacteric (strawberry, receptacle-derived) and climacteric (tomato, ovary-derived) fruits, depicting three key developmental stages: growth (cell division and expansion), ripening (metabolism of quality-related compounds), and senescence (nutrient remobilization and cellular breakdown). Arrows indicate key hormonal signals and associated PTM types at different stages—among which, Phospho, UBQ, and ROX have been reported to drive stage transitions, while novel PTMs remain underexplored. The right panel outlines future research directions focusing on PTM-regulated proteins, pathways, and networks, emphasizing the integration of exogenous and endogenous signals, stress responses, and precision improvement for balancing tolerance and quality. The parts marked in green are already reported studies. (B) Dual-pathway PTM regulation of metabolism and transport in fruit cells. Environmental cues (light, temperature, water, nutrients) and biotic factors (microorganisms, insects) are perceived by membrane receptors (potentially regulated by PTMs themselves), triggering endogenous signals (hormones, sugars, peptides, ROS, Ca^2+^). Core regulatory mechanisms include the following: (1) Indirect pathway (nuclear events): endogenous signals induce PTMs (e.g. phosphorylation, ubiquitination) of transcription factors (TFs) in the nucleus. Novel PTMs in this process warrant particular attention, as they may reveal new regulatory mechanisms. Modified TFs subsequently regulate the expression of genes encoding metabolic enzymes, hydrolases, and transporters. (2) Direct pathway (pan-cellular action): signals directly trigger PTMs of metabolic enzymes, hydrolases, and transporters across subcellular compartments (chloroplast, mitochondrion, vacuole, apoplast). This modulates transmembrane transport of sugars, water, and organic acids, and maintains the dynamic balance of metabolites (e.g. chloroplast pigments, mitochondrial VOCs, vacuolar anthocyanins). (3) Feedback regulation loop: metabolites (e.g. sugars) can feedback-regulate the endogenous signal pathway, forming a dynamic regulatory loop of “signal input → regulatory response → product feedback,” ensuring precise control over fruit development and quality formation.

Figure 6 presents an integrated roadmap for PTM-mediated fruit development regulation. Figure 6A illustrates the PTM landscape across developmental stages (growth, ripening, senescence) in both fruit types, highlighting established mechanisms alongside underexplored PTM types—SUMOylation, lactylation, and crotonylation—requiring investigation. Figure 6B depicts dual regulatory logic: indirect regulation via nuclear transcription factor modifications establishing transcriptional potential, and direct regulation modulating metabolic enzymes, transporters, and hydrolases for real-time adjustment. This model integrates environmental signals, hormonal cues, and metabolic feedback loops, illustrating how PTMs create a robust, multilayered control system.

Integrative research directions must move beyond cataloging individual modifications to understanding network properties. Multiscale integration should connect molecular PTM events to tissue-level coordination and whole-fruit developmental timing. Metabolic–PTM integration represents another frontier: metabolites like acetyl-CoA, ATP, and SAM serve as cofactors for PTM enzymes, directly linking cellular metabolism to protein modification status. Characterizing these metabolite–PTM feedback loops could reveal nutrient sensing mechanisms adjusting developmental timing. Computational modeling offers powerful approaches: machine learning trained on multiomics datasets could predict modification states, identify rate-limiting regulatory nodes, and simulate ripening outcomes under different scenarios, transforming fruit biology from descriptive to hypothesis driven.

Agricultural translation requires bridging molecular insights to field performance. As detailed in the “Methodological frontiers” section, precision editing of PTM sites offers unprecedented opportunities for trait improvement. The critical challenge is predicting which modifications will deliver desired phenotypes under field conditions. This requires integrating molecular understanding with agronomic performance data across diverse environments. Modifications conferring stress tolerance in controlled conditions may show minimal field effects where multiple stresses interact. Bridging this laboratory-to-field gap requires multilocation trials with environmental monitoring to identify interactions between PTM modifications and environmental conditions—revealing whether “universal PTMs” exist that improve traits regardless of environment, or whether context-specific engineering is necessary.

Climate change elevates PTM research urgency. Rising temperatures, erratic precipitation, and increased pest pressure demand fruits with enhanced stress resilience while maintaining quality and yield. PTM networks integrating environmental stress signals with developmental progression represent ideal targets for breeding climate-adapted varieties. Redox modifications linking oxidative stress to ripening control could enable varieties that slow ripening under stress, then resume when conditions improve. Phosphorylation cascades integrating temperature signals could generate heat-tolerant varieties maintaining productivity during heat waves.

The ultimate vision is comprehensive, predictive understanding of fruit ripening as an integrated PTM network, enabling rational design through targeted modifications. The convergence of technologies described in the “Methodological frontiers” section—high-resolution PTMomics, single-cell profiling, AI-guided prediction, and precision editing—enables hypothesis-driven PTM engineering guided by predictive models. Computational approaches modeling PTM networks as dynamic systems could enable *in silico* evaluation before experimental validation, dramatically accelerating improvement cycles.

Success will be measured not only by mechanistic insights, but also by delivering improved fruits with optimized nutrition, extended storage life, and stress resilience—attributes increasingly critical as agriculture confronts global change. The post-translational regulatory code of fruit ripening, once fully deciphered, could fundamentally transform both our understanding of plant development and our capacity to engineer better crops. PTM-based breeding approaches could deliver fruits that maintain quality under stress, minimize postharvest losses, and provide enhanced nutrition—contributions extending to global food security and human health [159–161].
